# Metabolomic Analysis in Neurocritical Care Patients

**DOI:** 10.3390/metabo13060745

**Published:** 2023-06-11

**Authors:** Maged Kharouba, Dimple D. Patel, Rami H. Jaber, Sherif Hanafy Mahmoud

**Affiliations:** Faculty of Pharmacy and Pharmaceutical Sciences, University of Alberta, Edmonton, AB T6G 2E1, Canada; mkharoub@ualberta.ca (M.K.); dimple@ualberta.ca (D.D.P.); rjaber@ualberta.ca (R.H.J.)

**Keywords:** metabolomics, neurocritical care, subarachnoid hemorrhage, traumatic brain injury, stroke, intracerebral hemorrhage

## Abstract

Metabolomics is the analytical study of metabolites in biological matrices using high-throughput profiling. Traditionally, the metabolome has been studied to identify various biomarkers for the diagnosis and pathophysiology of disease. Over the last decade, metabolomic research has grown to include the identification of prognostic markers, the development of novel treatment strategies, and the prediction of disease severity. In this review, we summarized the available evidence on the use of metabolome profiling in neurocritical care populations. Specifically, we focused on aneurysmal subarachnoid hemorrhage, traumatic brain injury, and intracranial hemorrhage to identify the gaps in the current literature and to provide direction for future studies. A primary literature search of the Medline and EMBASE databases was conducted. Upon removing duplicate studies, abstract screening and full-text screening were performed. We screened 648 studies and extracted data from 17 studies. Based on the current evidence, the utility of metabolomic profiling has been limited due to inconsistencies amongst studies and a lack of reproducible data. Studies identified various biomarkers for diagnosis, prognosis, and treatment modification. However, studies evaluated and identified different metabolites, resulting in an inability to compare the study results. Future research towards addressing the gaps in the current literature, including reproducing data on the use of specific metabolite panels, is needed.

## 1. Introduction

Metabolomics, the study of metabolites in biological matrices, is a crucial tool for identifying biomarkers and developing individualized treatment plans [1]. It also has the potential to reveal the pathophysiology of disease states through the detection of systemic changes in metabolite levels, followed by identification of the metabolic pathways involved. Over the past decade, untargeted metabolomics research has uncovered numerous pertinent biomarkers linked to various biological systems’ complex phenotypes [2]. This has further helped predict individual patient outcomes and optimize treatment targets. Additionally, metabolomics research has also advanced in the field of developing efficient and faster diagnostic techniques as compared to traditional ones. There is growing evidence supporting the potential utility of metabolomic profiling in acute illness, including patients with life-threatening neurological illnesses (neurocritical care population). In this review, we summarize the available evidence regarding the use of metabolomic profiling in neurocritically ill patients during the acute phase. Furthermore, we compile an overview of the various objectives of metabolomic profiling. These encompass the development of diagnostic tests, the determination of underlying pathophysiology and differences among populations, and the establishment of a metabolite panel for prognostic evaluation and risk stratification. Specifically, we evaluated the current literature in aneurysmal subarachnoid hemorrhage (aSAH), traumatic brain injury (TBI), and intracranial hemorrhage (ICH), the most common illnesses encountered in neurocritical care. This review will uncover areas of knowledge deficiency and provide direction for future research on metabolomic profiling.

## 2. Literature Search Strategy

A literature search of databases of available evidence pertaining to metabolome profiling in life-threatening neurological conditions in humans was conducted. Databases searched included Medline and EMBASE from inception to 7 June 2022. Keywords utilized were [(“subarachnoid hemorrhage” OR “SAH” OR “intracerebral brain hemorrhage” OR “cerebral hemorrhage” OR “hemorrhagic stroke” OR “stroke” OR “traumatic brain injury” OR “TBI” OR ”meningoencephalitis” OR “status epilepticus”) AND (“metabolomics” OR “metabolome”)]. After the removal of duplicates, the remaining studies underwent title and abstract screening. Studies on neurological conditions outside of the interest of this review and studies analyzing metabolome profiling outside of the acute phase were excluded. Nonhuman and non-English studies that could not be easily translated into English, as well as review articles, summary resources, commentaries, or editorials, were excluded.

## 3. Results of Literature Search

**Figure 1:**
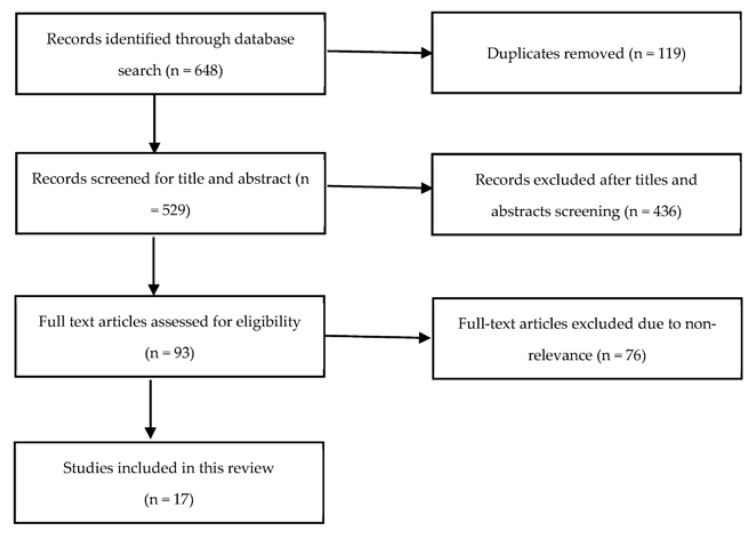
Flow diagram of the literature search for studies addressing metabolome profiling in neurocritical care population.

As depicted in Figure 1, after conducting a comprehensive literature search, we identified a total of 648 relevant studies. After the removal of duplicate studies, 529 studies remained and underwent title and abstract screening. Finally, after the completion of full-text screening, 17 studies were included in this review: 6 in aSAH, 10 in TBI, and 1 in ICH. Articles for status epilepticus and meningoencephalitis were also searched but did not yield any result. Table 1 summarizes the studies included in this review. All of the studies were observational: 14 prospective and 3 retrospective. The biological samples utilized varied across studies. A total of 10, 1, and 5 studies utilized blood, urine, and cerebrospinal fluid (CSF) samples, respectively. Two studies in TBI utilized cerebral microdialysates. The majority of the metabolomic profiling was untargeted (14 studies) and three studies were targeting specific metabolite profiles. A total of five studies used validation and discovery groups in their studies. Table 2 summarizes the key findings obtained from the reviewed studies.

## 4. Discussion

Metabolome profiling has been evaluated for a variety of applications in neurocritical conditions. Emerging data support the potential utility of metabolomic analysis in diagnosis and differential diagnosis, prognosis and functional outcome prediction, identification of underlying pathophysiological pathways, and many other clinical implications.

### 4.1. Aneurysmal Subarachnoid Hemorrhage (aSAH)

aSAH is a serious medical condition resulting from bleeding in the subarachnoid space secondary to a ruptured intracranial aneurysm. aSAH can have a significant burden on patients, families, and society. The mortality rate for aSAH is high, with up to 50% of patients dying within the first few weeks after onset. Among those who survive, many may experience long-term disabilities, such as cognitive impairment and motor deficits. Despite advancements in research, the pathophysiology of aSAH remains uncertain and clinical outcomes remain poor [3,4,5]. A thorough understanding of the pathophysiological pathways following aSAH is, therefore, needed. Metabolomic studies conducted in aSAH patients aimed to identify metabolic changes that occur as a result of the condition and to gain insights into its pathophysiology and potential biomarkers for diagnosis, prognosis, and treatment. Six metabolomic profiling studies have been reported in aSAH patients. Metabolomic studies in aSAH have used various analytical techniques, such as nuclear magnetic resonance (NMR) spectroscopy and mass spectrometry, to profile metabolites in cerebrospinal fluid (CSF) and blood samples from patients. These studies have identified alterations in various metabolic pathways, including amino acid metabolism and lipid metabolism, among others. Overall, the primary goal of all the reported studies was to utilize metabolome profiling during the acute phase to predict prognostic outcomes (Table 1).

A prospective case–control study by Dunne et al., 2004 reported that elevated lactate and glutamine concentrations as well as decreased glucose concentration were associated with Hunt and Hess (H&H) grade, Glasgow Coma scale (GCS), measures of disease severity, and with the cognitive outcome score (COS), a measure of patient outcomes [3]. Elevation in the ratio of lactate-to-glucose had a statistically significant association with temporal progression of aSAH. Additionally, these metabolites also helped differentiate blood-contaminated CSF from causes other than aSAH. Therefore, this study is unique as it identifies the potential use of 1H-NMR data to confirm the diagnosis of aSAH. Overall, the pattern recognition models generated using 1H-NMR data predicted cognitive outcomes and presence of aSAH. Lastly, elevated glutamine level is indicative of greater neuronal inactivity in aSAH patients as compared to controls. This is likely due to the activity of glutamine synthase found in compartmentalized glial cells [20]. The enzyme deactivates excitatory glutamate by converting it into glutamine and releasing it into the extracellular fluid to further replenish neuronal glutamate stores.

Two studies investigated the potential association between metabolites and modified Rankin Scale (mRS) at discharge and at 90 days, a common functional outcome scale in stroke studies. They analyzed samples collected on days 0–5, 6–10, and 11–14 post-bleeding utilizing LC/MS [4,5]. Koch et al., 2021 reported that elevations in CSF levels of symmetric dimethylarginine (SDMA), dimethylguanidine valeric acid (DMGV), and ornithine are significantly associated with poor mRS at discharge and at 90 days. This is likely because these vasoactive molecules are linked to nitric oxide that predicts poor outcome after severe aSAH [21]. Additionally, a statistically significant elevation of SDMA was reported in the CSF of aSAH patients as compared to non-aneurysmal aSAH controls. The pathophysiological mechanism underlying this systemic elevation remains unclear. On the other hand, Stapleton et al., 2019 reported that elevated plasma taurine levels following aSAH predict a favorable mRS (0–2) at 90 days following aSAH bleeding. The results remained consistent upon performing univariate and multivariate logistic regression analyses, adjusted for age, H&H grade, modified Fisher grade, hydrocephalus and delayed cerebral ischemia. This elevation in taurine can be attributed to downregulation of pro-inflammatory cytokines as seen in experimental animal studies [22]. Another plausible mechanism highlights the upregulation of taurine after cerebral stress for neuroprotection [23,24]. Despite the similarities in study design with Koch et al., 2021 [4], caution should be exercised when interpreting data due to variability in metabolites of interest, likely because samples were drawn from different biological fluids, i.e., CSF vs. plasma sample. In addition, relying on CSF samples could potentially bias the cohort study towards higher-grade aSAH patients. Furthermore, in Stapleton et al., less than 30% of plasma samples were drawn and analyzed during days 0–5, thereby decreasing the power of analysis.

In the retrospective cohort study by Sjöberg et al., 2015, venous blood samples were analyzed using GC/MS [6]. The study concluded that myo-inositol was elevated on day 7 after aSAH, and was significantly and positively correlated with good GOS (4–5) at 1-year follow-up. The study is unique as no other studies analyzed the effects of myo-inositol on aSAH outcomes. Myo-inositol is a critical metabolite for three reasons. First, activation of endothelin receptor type A during the final step of the metabolism of myo-inositol to inositol can cause an increase in intracellular calcium and vasoconstriction in cerebral smooth muscle cells [25]. Second, experimental studies in aSAH patients revealed an inositol 1,4,5-triphosphate-dependent elevation in calcium levels in human astrocytes in response to CSF exposure. Consequently, mitochondrial permeability gradually rises and causes cell necrosis [26]. Finally, myo-inositol functions as a potent osmolyte, countering the effects of cytotoxic and vasogenic edema while decreasing the pressure gradient across the blood–brain barrier [27,28].

Prospective cohort studies by Li et al., 2019 and Lu et al., 2018 analyzed CSF samples using GC/TOF-MS [7,8]. Li et al., 2019 performed additional analysis utilizing LC/TOF-MS and noted that aSAH altered the CSF metabolome involving carbohydrate, lipid and amino acid metabolism [7]. The authors noted a statistically significant and positive correlation between pyruvate metabolism and aSAH patients with high disease severity (H&H and WFNS > 3). Additionally, the authors concluded that aSAH patients with unfavorable outcomes presented with upregulated CSF amino acid levels and enhanced lipid biosynthesis. Li et al., 2019 identified that the concentrations of aspartate, asparagine, methionine, phenylalanine, tryptophan, leucine, isoleucine, ornithine, tyrosine, phenylpyruvate, serine, glycine, threonine, valine, alanine, histidine, 3-phospho-serine, homoserine, homocysteine, homocysteic acid, glutamate, glutamine, and ornithine are higher in the CSF of patients with a lower GOS score due to the significant enhancement of amino acid biosynthesis and lipid metabolism in aSAH patients. The findings from the study have clinical implications in predicting aSAH severity and unfavorable outcomes at 1-year post-SAH. Similarly, Lu et al., 2018 analyzed 16 metabolites, primarily free amino acids [8]. The study concluded that six metabolites (2-hydroxyglutarate, tryptophan, glycine, proline, isoleucine, and alanine) correlated with GOS at 1-year post-aSAH independent of vasospasm status. Additionally, four of these metabolites (tryptophan, proline, isoleucine, and alanine) significantly increased from time at admission to time during hospitalization. Therefore, such untargeted metabolomic research can help identify a panel of metabolites associated with disease prognosis.

In conclusion, with emerging evidence, metabolomics profiling can be utilized to predict long-term outcomes and cognitive impairment in aSAH patients. However, caution should be exercised when interpreting current data due to inter-variability in metabolites identified and analyzed in these studies. Further studies should be conducted to replicate the results of current studies and address the limitations of study designs as identified.

### 4.2. Traumatic Brain Injury (TBI)

TBI is a major cause of death and disability in young adults worldwide [29]. By 2031, TBI is expected to be the most common neurological condition affecting Canadians, in addition to Alzheimer’s disease and other dementias, as well as epilepsy [30]. Injury is classified into two main stages: primary and secondary. Primary injury is caused by direct penetrating or non-penetrating insults caused by mechanical forces, such as acceleration/deceleration, rotational forces, and blast and blunt injuries [29,31]. Consequently, these forces damage glial cells, neurons, and vasculature in a focal, multifocal, or diffuse pattern [29]. Secondary injury following TBI is likely due to ischemia, vasogenic edema, cytogenic edema, inflammation, and formation of reactive oxygen species [31,32]. TBI is additionally classified into mild, moderate and severe TBI employing the GCS scoring system [31]. Over the last decade, the applicability of metabolome profiling in TBI has expanded towards the identification of diagnostic biomarkers, classification of disease severity, predicting patient outcomes by identifying prognostic biomarkers, and treatment optimization.

#### 4.2.1. Identification of Diagnostic Biomarkers

Two prospective studies correlated the metabolomic profiles with brain imaging findings following TBI. Thomas et al., 2020 performed untargeted metabolomic analysis utilizing GC-TOFMS [9]. Serum samples were collected 12 h after admission. The goal of the study was to determine whether a correlation exists between structural MRI (sMRI) findings and circulating serum metabolites, following TBI. Metabolites were summarized into eight clusters based on their chemical identity and sMRI data were reduced to 15 independent components (ICs). Partial correlation analysis indicated a significant association between four metabolite clusters and specific ICs, reflecting both the gray and white matter brain injury. Metabolic cluster 8, primarily consisting of amino acids, showed correlations with supra- and infratentorial white matter areas. This suggests that the metabolites in this cluster could be indicative of white matter injury. Metabolic clusters 3 (sugar intermediates), 4 (fatty acids) and 7 (amino acids, microbial metabolites, and sugar intermediates) were associated with changes to cortical gray matter regions. Cluster 7, consisting of erythronic acid, was also correlated to the brain region closer to the brain stem; the right fusiform gyrus. Furthermore, two metabolites, myo-inositol and erythronic acid, along with neurofilament light polypeptide (NF-L), discriminated between positive and negative sMRI findings. Myo-inositol was significantly elevated in severe and moderate TBI. However, the mechanism of action of myo-inositol is largely unknown. Some studies show that myo-inositol is likely related to changes in glial cells or osmolarity following TBI [33,34]. Similar to what was described in aSAH, myo-inositol’s osmolytic properties can contribute to the regulation of brain edema and assist in maintaining cell volume homeostasis. Similarly, erythronic acid is increased in TBI, likely due to dysregulation of the pentose–phosphate pathway [35,36]. The study’s future implications involve utilizing metabolomic profiling to predict changes in brain morphology by increasing the evidence related to the diagnostic and prognostic value of metabolites. The study was limited due to differences in timing of collection of blood samples and MRI scan. Similarly, Dickens et al., 2018 performed untargeted metabolomic analysis using GC-TOFMS [10]. Plasma samples were collected within 12 h of admission for TBI. The authors reported that combinations of decreased 2-aminobutyric acid levels and increased acetoacetic acid, pentitol, inositol, and ribonic are able discriminate between patients with intracranial abnormalities on CT and patients with a normal CT. Furthermore, a set of another 3 metabolites including 2-hydroxybutyric acid, isovaleryl glucuronide and phenolic compound was identified to differentiate TBI patients with diffuse injury from those with mass lesion. The exact mechanism of action of these metabolites and their implications in the pathophysiology of TBI is largely unknown. If further studies confirm the diagnostic value of these metabolic biomarkers, they can help prevent unnecessary CT scans, reduce the cost of diagnostics and reduce the radiation load.

As opposed to untargeted analysis, Fiandaca et al., 2018 performed a targeted six-metabolite panel analysis utilizing tandem MS/MS to objectively classify college athletes sustaining moderate TBI from non-injured teammates within 6 h of trauma [11]. The six-metabolite panel included 2-hydroxypalmitic acid (FA 2-OH C16:0), stearic acid (FA C18:0), tauroursodeoxycholic acid (TUDCA), phosphatidylethanolamine plasmalogen (PE ae C36:4), diacyl-phosphatidyl-ethanolamine (PE aa C38:6) and lyso-phosphatidylcholine (LysoPC a C20:4). A decreased level of FA 2-OH C16:0, TUDCA, and PE aa CC38:6 was observed in TBI patients, whereas metabolites FA C18:0, PE ae C36:4, and LysoPC a C20:4 increased. Future studies analyzing this metabolite panel in a different patient population are required to confirm the diagnostic potential of the panel. The implications of such studies include faster diagnosis, especially in areas with minimal access to imaging technology.

#### 4.2.2. Identification of Prognostic Biomarkers and Markers of Disease Severity

Two studies conducted metabolomic analysis in cerebral microdialysates. Cerebral microdialysis is an emerging technique used to measure the chemical composition of the extracellular fluid in the brain. In neurocritical care, cerebral microdialysis is used to monitor the metabolic and biochemical changes that occur in the brain after brain injury. By analyzing the chemical composition of the extracellular fluid, clinicians can assess the extent of brain damage, monitor the effectiveness of treatments, and detect complications such as cerebral edema or ischemia. Orešič et al., 2016 sampled serum and cerebral microdialysates from patients with mild, moderate and severe TBI, and performed untargeted metabolic analysis [12]. The samples were obtained within 12 h of hospital admission. Two medium-chain fatty acids (octanoic acid (OA) and decanoic acid (DA)) and several sugar metabolites including 2,3-bisphosphoglyceric acid were elevated in the serum samples of TBI patients. These metabolites were associated with poor outcomes in TBI patients at 3 months following injury. The results were further validated in brain microdialysates sampled from 12 severe TBI patients. OA and DA are significant metabolites because they have been reported to cause mitochondrial dysfunction through uncoupling and inhibiting oxidative phosphorylation. Several studies have also shown that DA and OA play a role in brain energy metabolism and elicit lipid and protein oxidative damage [37,38,39,40]. Eiden et al., 2019 also analyzed cerebral microdialysates to identify cerebral metabolic states that were associated with GOS at 6 months [13]. This is amongst the first studies to suggest the importance of metabolome profiling in improving clinical outcomes following TBI through nutritional modifications during ICU stay. Although promising, the utility of cerebral microdialysis is limited by its availability in treatment centers.

Utilizing urine samples for metabolomic analysis is more feasible and readily available compared to cerebral microdialysis and CSF sampling. Bykowski et al., 2021 utilized 1H-NMR to analyze urine samples from patients with TBI drawn at 7 days and 6 months post-injury [14]. Homovanillate, L-methionine, and thymine decreased significantly in TBI patients. This decrease in metabolites was associated with worse patient outcomes and TBI severity as assessed using the Montreal cognitive Assessment (MoCA) and Functional Independence Measure (FIM). Homovanillate is a major dopamine metabolite and hence has the potential to act as an indirect indicator of diminished circulating dopamine. TBI patients have been demonstrated to have reduced binding to dopamine transporters in the striatum, explaining the damage to striatal areas [41]. L-methionine is implicated in angiogenesis and vascular remodeling processes stimulated by TBI [42]. Prior studies have shown correlation between decreased blood methionine levels and increased injury severity [18]. Increased urinary thymine levels may indicate dihydrothymine dehydrogenase deficiency, an enzyme that catabolizes thymine to beta-aminoisobutyric acid that down-regulates the production of proinflammatory cytokines. Thus, increased levels of thymine initially appear to be neuroprotective in the acute injury phase by suppressing inflammation after injury [43,44]. The study was limited by its small sample size, where only eight male TBI patients were included.

Glenn et al., 2013 employed 1H-NMR to conduct targeted metabolomic analysis on CSF samples from 44 individuals with severe TBI as well as 13 control subjects, with the objective of establishing a metabolomic fingerprint for TBI [15]. Ten metabolites analyzed in the study included β-glucose, lactate, propylene glycol, glutamine, alanine, α-glucose, pyruvate, creatine, creatinine and acetate. Statistically significant increase in propylene glycol and decrease in total creatinine was noted in TBI patients. Reduced total creatinine could be attributed to augmented renal clearance observed in TBI patients leading to increased creatinine clearance [45,46,47]. Additionally, univariate generalized models demonstrated that propylene glycol, glutamine, α-glucose, creatinine were predictors of changes observed in cerebral metabolic rate of oxygen, intracranial pressure and extended GOS at 6 months. However, the underlying pathophysiology of severe TBI resulting in these prognostic outcomes remains unclear.

Another prospective case–control study conducted by Thomas et al., 2022 in 716 TBI patients and 229 controls further analyzed serum metabolome to classify disease severity and predict prognosis [16]. They noted that choline phospholipids (lysophosphatidylcholines, ether phosphatidylcholines and sphingomyelins) were inversely associated with TBI severity. Pathway analysis suggested that lipid, sugar and amino acid pathways were amongst the most significantly affected pathways in TBI patients. The large sample size of the study and adding validation groups instilled further confidence in the study results. Comparable to results from Thomas et al., 2022, a study conducted by Martha et al., 2022 on plasma samples of TBI patients using LC/MS noted statistically significant increase in phosphatidylcholine and decrease in phosphatidylethanolamine [16,17]. The authors proposed that the increase in phospholipids could be attributed to the repair of compromised phospholipid membranes and an early response to TBI. The study identified phatidylinositol-3,4,5-trisphosphate as a metabolite of significance as it was associated with improved outcomes in older adults with moderate TBI, as assessed on extended GOS at 3- and 6 months. However, the authors did not assess the underlying pathophysiology for this association. Furthermore, the study results cannot be generalized to a larger population as it primarily focused on male Caucasian older adults diagnosed with mild TBI.

#### 4.2.3. Treatment Optimization

Metabolomic profiles were explored as indicators for management optimization in TBI patients in four studies. Dash et al., 2016 conducted targeted analysis of methionine and its metabolites using LC/MS or GC/MS [18]. Plasma samples were collected within 24 h of injury. Patients with severe TBI were shown to have decreased levels of methionine, s-adenosylmethionine, betaine and 2-methylglycine. This is likely due to decreased metabolism of methionine through the transmethylation cycle. Additionally, patients with moderate TBI also showed decreased levels of methionine, α-ketobutyrate, 2 hydroxybutyrate and glycine, albeit to lesser degrees than those detected in the severe TBI group. These findings further support the findings by Eiden et al., 2019 and Bykowski et al., 2021 [13,14]. Taken together, methionine supplementation in TBI patients during the acute injury phase could potentially improve disease prognosis. Currently, only a few clinical studies are available assessing the potential benefits of intravenous methionine supplementation [48,49,50]. However, Dash et al., 2016 did not account for variability in plasma methionine concentrations due to food source, a potential limitation of the study. The study also noted significantly decreased levels of cysteine, glycine, several gamma-glutamyl amino acids and 5-oxoproline in severe TBI patients. Cysteine and glycine are important precursors for glutathione, a major antioxidant molecule. Decreased levels of glutathione have been implicated in increased brain damage as they increase vulnerability to oxidative stress [51], especially during the high metabolic state of severe TBI patients. Therefore, Dash et al., 2016 further anticipated the role of glutathione supplementation in providing neuroprotection. Lastly, the transportation of gamma-glutamyl amino acids into cytosol to yield free amino acids and 5-oxoproline is dependent on glutathione. Reduced intracellular transport via gamma-glutamyl cycle can further reduce the intracellular availability of methionine and other free amino acids [52].

In conclusion, most metabolomic studies in TBI focused on classifying the prognostic markers and disease severity of TBI. There is an emerging interest in analyzing the diagnostic potential of metabolites. However, future studies should focus on validating the results of the metabolites studied with a larger patient population to account for the inherent variability in patient characteristics. Future studies should also aim to conduct multivariate statistical analysis to establish confidence in the correlational analysis between metabolites and disease severity. Lastly, multiple studies have established the correlation between methionine and disease severity of TBI. Further studies focusing on nutritional interventions and impact on prognosis should be considered. 

### 4.3. Intracranial Hemorrhage (ICH)

ICH is a result of spontaneous rupture of small cerebral arteries due to hypertensive arteriopathy or cerebral amyloid angiopathy [53,54]. ICH accounts for 15% of strokes and the loss disability-adjusted life years caused by hemorrhagic stroke is significantly higher than ischemic stroke [55]. There is very limited evidence available on metabolome profiling in ICH patients. Future studies should focus on expanding the scope of research in determining the utility of metabolomics in this patient population. Our search resulted in one study related to metabolome profiling in ICH patients. Sun et al., 2021 performed non-targeted metabolomic analysis on serum samples obtained from ICH patients [19]. The study identified 11 metabolites of significance that are associated with multiple pathophysiological pathways following ICH. Those pathways include sphingolipid and phospholipid metabolism, fatty acid oxidation, pro-inflammatory state and oxidative stress. The 11 metabolites included L-carnitine, L-octanoylcarnitine, phytosphingosine (PHS), lysophosphatidylcholine (lysoPC) (14:0/0:0), lysophosphatidyl ethanolamine (lysoPE) (0:0/18:2), lysoPC (18:3), lysoPC (20:5/0:0), phosphatidylcholine (PC) (14:1/18:1), phosphatidyl ethanolamine (PE) (22:4/P-18:0), lactosylceramide (d18:1/12:0), and PC (20:3/22:6). Furthermore, L-carnitine and phosphatidylcholine (20:3/22:6) were able to discriminate ICH patients from controls. Overall, the study findings have implications in determining the underlying pathophysiology of ICH and identification of diagnostic biomarkers.

## 5. Conclusions and Future Directions

Metabolomic profiling has multiple applications in neurocritical care. For instance, multiple studies identified the correlation between metabolite concentration and functional outcomes for aSAH. However, the study results have not been reproduced in different populations using different analysis techniques to ensure a true correlation. Similarly, metabolomic profiling has its applicability in the classification of TBI severity, diagnostics and functional outcomes. The studies included in this review analyzed different metabolites and metabolic pathways. This review is limited in its ability to provide concrete evidence on the clinical implications of a specific metabolite due to a lack of reproducible data. Furthermore, metabolomics is an emerging field of medicine and thereby, the studies available are limited in multiple ways. Some common limitations of the studies include smaller sample size, narrow inclusion criteria, a lack of validation of methods of analysis, insufficient clinical information available for comparison, and difficulty in obtaining patient samples such as CSF and cerebral microdialysates that represent the metabolome closer to the site of injury. Despite the limitations, this review identifies gaps in the literature and provides direction for future studies. We recognize the need for metabolomic research in personalized medicine to improve diagnostic tests, determine underlying pathophysiology, predict prognostic outcomes, and optimize treatment targets. Future studies should focus on reproducing these study outcomes and utilizing multiple statistical models to avoid biased results. The utilization of multi-omic approaches and animal studies in concert with metbolomic studies will provide valuable insights on the understanding of disease mechanisms, identifying potential biomarkers, and discovering novel therapeutic targets.

## Figures and Tables

**Table 1:** Summary of the studies included in this review.

Study	Study Design	n	Age (Years)	Females n (%)	Method of Analysis	Sample Type	Targeted/Untargeted	Metabolite Number	Sample Taken	Metabolite of Significance	Concentration	Main Findings
aSAH												
Dunne et al., 2004 [3]	Prospective cohort	16	Range 16–68	11 (69)	1H NMR	CSF	Untargeted	~60	3, 4, 5, 6, 9, 10, and 12 days post-bleeding	Lactate and glutamine Glucose	Elevated Reduced	Associated with H&H, GCS, and COS
Koch et al., 2021 [4]	Retrospective cohort	81	58 ± 12	49 (60)	LC/MS	CSF	Untargeted	138	0–5, 6–10, 11–14 days post-bleeding	Ornithine, SDMA, and DMGV	Elevated	Statistically significant association with poor mRS (3–6) at discharge and at 90 days post-SAH
Stapleton et al., 2019 [5]	Prospective cohort	137	56 ± 12	93 (68)	LC/MS	Plasma	Untargeted	163	0–5, 6–10, and 11–14 days post-bleeding	Taurine	Elevated	Statistically significant association with favorable mRS (1–2) at 90 days post-SAH
Sjoberg et al., 2015 [6]	Retrospective cohort	50	Median 59 (26–82)	35 (70)	GC/MS	Serum	Untargeted	59	1–3 and 7 days post-bleeding	Myo-inositol on day 7	Elevated	Significantly associated with favorable GOS at 1-year follow up
Li et al., 2019 [7]	Prospective cohort	46	61 ± 11	28 (61)	GC-TOF MS and LC –TOF MS	CSF	Untargeted	39	Within the first 7 days post-bleeding	Pyruvate metabolites Amino acid levels and lipid biosynthesis	Elevated	Significantly associated with aSAH severity evaluated with H&H and WFNS scales
Lu et al., 2018 [8]	Prospective cohort	15	56 ± 17	11 (73)	GC-TOF MS	CSF	Untargeted	97	Day 0 and within the anticipated vasospasm timeframe	Six free amino acids (2-HG, Trp, Gly, Pro, Iso, and Ala)	Decreased	Significantly associated with favorable GOS at 1-year follow up
TBI												
Thomas et al., 2020 [9]	Prospective cohort	96	49 ± 19	33 (34)	GC-TOF MS	Serum	Untargeted	451 in 8 clusters	Within 12 h from admission	Metabolite cluster alterations Myo-inositol and erythronic acid	Alterations Elevated	Correlated with structural MRI findings Discriminated positive and negative MRI findings for TBI diagnosis
Dickens et al., 2018 [10]	Prospective cohort	210	Range 18–91	58 (28)	GC-TOF MS	Serum	Untargeted	36	Within 12 h from admission	2-aminobutyric acid acetoacetic acid, pentitol, inositol, and ribonic acid	Elevated Decreased	Metabolites were able to discriminate between patients with intracranial abnormalities on CT and patients with a normal CT
Fiandaca et al., 2018 [11]	Prospective cohort	62	Range 18–23	31 (50)	Tandem MS/MS	Plasma	Targeted	Six-metabolite panel	Within 6 h of injury	FA C18:0, PE ae C36:4, LysoPC a C20:4 panels FA 2-OH C16:0, TUDCA, PE aa CC38:6 panels	Elevated Decreased	Help in the classification of moderate TBI from control (no injury)
Orešič et al., 2016 [12]	Prospective cohort	266	Range 18–91	86 (32)	GC-TOF MS	Serum and brain microdialysate	Untargeted	465	Within 12 h from admission	Two medium-chain fatty acids (decanoic and octanoic acids) and sugar derivatives including 2,3-bisphosphoglyceric acid	Elevated	Metabolites were associated with poor outcomes at 3 months
Eiden et al., 2019 [13]	Prospective cohort	38	44 ± 17	23 (61)	LC-MS	Brain microdialysate	Untargeted	40	Samples were collected every 60 min	Metabolite patterns associated with ketometabolism	Altered	Cerebral metabolic states that were associated with GOS at 6 months
Bykowski et al., 2021 [14]	Retrospective cohort	8	45 ± 18	0 (0)	1H-NMR	Urine	Untargeted	134	7 days and 6 months postinjury	Homovanillate, L-methionine, and thymine	Decreased	This decrease in metabolites was associated with worse patient outcomes and TBI severity
Glenn et al., 2013 [15]	Prospective cohort	57	38 ± 15	12(21)	1H-NMR	CSF	Targeted	10 metabolite targets	NR	Propylene glycol, gluta-mine, α-glucose, and creatinine	Altered	Statistically significant increase in propylene glycol and decrease in total creatinine; propylene glycol, glutamine, α-glucose, and creatinine were predictors of changes observed in extended GOS at 6 months
Thomas et al., 2022 [16]	Prospective cohort	945	Range 27–80	335 (35)	UHPLC-QTOF-MS	Serum	Untargeted	30	Within 24 h of injury	Choline phospholipids (lysophosphatidylcholines, ether phosphatidylcholines, and sphingomyelins)	Altered	Metabolites were inversely associated with TBI severity
Martha et al., 2021 [17]	Pilot study using samples from parent prospective cohort study	24	67 ± 7	6 (25)	LC/MS	Plasma	Untargeted	2	0, 3, and 7 days and 1, 3, and 6 months postinjury	Phosphatidylcholine Phosphatidylethanolamine	Elevated Decreased	Pohosphatidylinositol-3,4,5-trisphosphate was associated with (GOS-E) at 3 and 6 months
Dash et al., 2016 [18]	Prospective cohort	60	Range 14–57	14 (23)	LC-MS or GC-MS	Plasma	Targeted	18	Within 24 h of injury	Severe TBI has decreased levels of methionine, s-adenosylmethionine, betaine, and 2-methylglycine	Decreased	Metabolites associated with disease severity
ICH												
Sun et al., 2021 [19]	Prospective cohort	60	Range 46–72	22 (37)	UPLC/Q-TOF	Serum	Untargeted	11	NR	L-caritine and phosphatidylcholine	Altered	Metabolites were able to discriminate ICH from controls

aSAH, subarachnoid hemorrhage; aSAH, aneurysmal subarachnoid hemorrhage; TBI, traumatic brain injury; ICH, intracerebral hemorrhage; n, number or patients enrolled; NMR, nuclear magnetic resonance; CSF, cerebrospinal fluid; COS, cognitive outcome score; H&H, Hunt and Hess scale; GCS, Glasgow coma scale; LC/MS, liquid chromatography/mass spectrometry; GC/QTOF-MS, gas chromatography/quadrupole time-of-flight mass spectrometry; LC/TOF-MS, liquid chromatography/time-of-flight mass spectrometry; mRS, modified Rankin scale; WFNS, World Federation of Neurological Surgeons Scale; GOS, Glasgow Outcome Scale; NR, not reported, MRI, magnetic resonance imaging; UHPLC, ultra-high-performance liquid chromatography.

**Table 2:** Summary of key findings from the reviewed studies.

Marker	Level	Disease	Result	Reference
Metabolites associated with disease severity				
Amino acids and lipids	↑	aSAH	Poor H&H and WFNS	[7]
Choline phospholipids (lysophosphatidylcholines, ether phosphatidylcholines and sphingomyelins)	Altered	TBI	TBI severity	[16]
FA C18:0, PE ae C36:4, LysoPC a C20:4 panels	↑	TBI	TBI severity classification	[11]
FA 2-OH C16:0, TUDCA, PE aa CC38:6 panels	↓	TBI	TBI severity classification	[11]
Glutamine	↑	aSAH	Poor H&H and GCS	[3]
Glucose	↓	aSAH	Poor H&H and GCS	[3]
Homovanillate, L-methionine, and thymine	↓	TBI	TBI severity	[14]
Lactate	↑	aSAH	Poor H&H and GCS	[3]
L-caritine and phosphatidylcholine	Altered	ICH	ICH severity	[19]
methionine, s-adenosylmethionine, betaine, and 2-methylglycine	↓	TBI	TBI severity	[18]
Pyruvate	↑	aSAH	Poor H&H and WFNS	[7]
Metabolites association with disease outcomes (potentially prognostic)				
Amino acids (2-HG, Trp, Gly, Pro, Iso, and Ala)	↓	aSAH	Favorable GOS	[8]
DMGV	↑	aSAH	Poor mRS	[4]
Glutamine	↑	aSAH	Poor COS	[3]
Glucose	↓	aSAH	Poor COS	[3]
Glutamine	Altered	TBI	Poor GOS	[15]
Homovanillate, L-methionine, and thymine	↓	TBI	Poor outcome	[14]
Lactate	↑	aSAH	Poor COS	[3]
Myo-inositol	↑	aSAH	Favorable GOS	[6]
medium-chain fatty acids (decanoic and octanoic acids)	↑	TBI	Poor outcome	[12]
Metabolite patterns associated with ketometabolism	Altered	TBI	Poor GOS	[13]
Ornithine	↑	aSAH	Poor mRS	[4]
Propylene glycol, gluta-mine, α-glucose, and creatinine	Altered	TBI	Poor GOS	[15]
Phosphatidylcholine	↑	TBI	Poor GOS	[17]
Phosphatidylethanolamine	↓	TBI	Poor GOS	[17]
Sugar derivatives as 2,3-bisphosphoglyceric acid	↑	TBI	Poor outcome	[12]
SDMA	↑	aSAH	Poor mRS	[4]
Taurine	↑	aSAH	Favorable mRS	[5]
Metabolites association with imaging findings (potentially diagnostic)				
2-aminobutyric acid	↑	TBI	CT	[10]
acetoacetic acid, pentitol, inositol and ribonic acid	↓	TBI	CT	[10]
Metabolite clusters	Altered	TBI	MRI	[9]
Myo-inositol and erythronic acid	↑	TBI	MRI	[9]

aSAH, aneurysmal subarachnoid hemorrhage; TBI, traumatic brain injury; ICH, intracerebral hemorrhage; COS, cognitive outcome score; H&H, Hunt and Hess scale; GCS, Glasgow coma scale; mRS, modified Rankin scale; WFNS, World Federation of Neurological Surgeons scale; GOS, Glasgow Outcome Scale; MRI, magnetic resonance imaging.
